# Outcome of a penetrating keratoplasty in a 3-month-old child with sclerocornea

**DOI:** 10.3205/oc000162

**Published:** 2020-08-07

**Authors:** Dominika Pohlmann, Mirjam Rossel, Daniel J. Salchow, Eckart Bertelmann

**Affiliations:** 1 Charité – Universitätsmedizin Berlin, corporate member of Freie Universität Berlin, Humboldt-Universität zu Berlin, and Berlin Institute of Health, Berlin, Germany

**Keywords:** congenital corneal opacity, graft survival, pediatric keratoplasty, sclerocornea

## Abstract

Sclerocornea is a rare congenital anomaly with clouding of the peripheral cornea that possibly extends up to the center of the cornea. Characteristically, a clear distinction (limbus) between sclera and cornea is lacking. Early surgical treatment is essential for preventing amblyopia, but penetrating keratoplasty in children carries a relatively high risk of complications. Especially for sclerocornea, penetrating keratoplasty has generally been reported to have a poor surgical outcome and a high risk of complications, including corneoscleral adhesions. Here, we report the 4-year follow-up on a child with sclerocornea, who was successfully operated on at the age of 3 months and had a favorable outcome. Our findings suggest that in some cases, penetrating keratoplasty may be an option to treat sclerocornea in young children.

## Introduction

A congenital corneal opacity occurs in 3 of 100,000 newborns [1] and may cause visual impairment and even blindness. In children, deprivation amblyopia may occur if a visually significant corneal opacity is not treated early on. A first report of a penetrating keratoplasty (PK) in a 9-month-old infant with sclerocornea was published in 1970 [2]. Still, PK in children is considered to carry a high risk for complications [2], [3], [4], [5], [6], [7], [8], and PK in sclerocornea is not recommended by some surgeons because of possible catastrophic results including iridocorneal adhesions and graft failure [2], [9]. The surgery is technically challenging in infants due to the elasticity of sclera and cornea, the shallow anterior chamber, anterior displacement of the lens-iris diaphragm, and the smaller size of the eye [10]. Difficult postoperative examinations and postoperative care contribute to a poor outcome compared to PK in adults. Postoperative complications include suture loosening, corneal infiltrates, and higher rates of graft failure [3], [6], [7], [10], [11], [12]. Repeated examinations under anesthesia may be required for postoperative management. Zhang et al. suggest that the main determinants for the outcome of PK in children include indication and types of surgery performed [10]. In their study conducted in Beijing, the most common indication for PK in children younger than 12 years was congenital corneal opacity which was not further specified. In the same study, the graft survival was 68.1% with a mean follow-up of 33.7 months in infants and children [10]. Graft failure occurred in 26 eyes of 79 (26.6%) in infants aged ≥3 months to 4 years [10]. Lin et al.’s study on children with congenital corneal opacities found that most eyes with clear grafts achieved ambulatory vision (≥20/960), especially children with bilateral opacity [13]. Of all surgical indications for PK in children, unilateral sclerocornea was associated with the worst visual outcome. Lin et al. proposed that sclerocornea might be associated with denser opacification and anterior segment dysplasia [13]; and average graft survival after PK for sclerocornea has been reported to be 36.4 months for children under 5 years [9]. Despite the concern connected with performing PK at an early stage, it may be considered and of value in select patients.

We report our case of a 3-month-old child with bilateral sclerocornea with 4-year follow-up after PK. 

## Case description

A 3-month-old girl presented in March 2015 with bilateral dense corneal opacity since birth. The family history was positive for anterior segmental mesenchymal dysgenesis. Her brother was born in 2019 and also showed corneal opacity, but only in one eye. The mother had a congenital cataract associated with a PITX3 gene mutation; the father had no pertinent ocular history. The children were not genetically examined.

**Figure 1:**
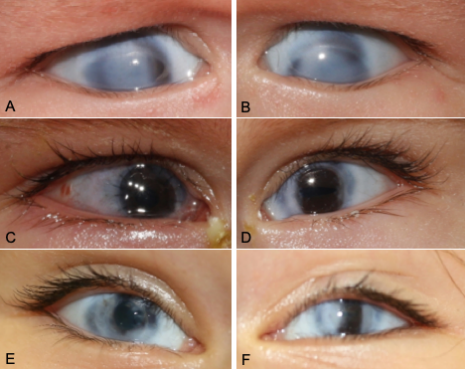
Pre- and postoperative stages of penetrating keratoplasty (PK) of the presenting case. The right (A) and left eye (B) revealed a congenital corneal opacity with scleral tissue extension in the center and the periphery of the cornea, and discreet limbal vascularisation. The histological findings confirmed sclerocornea. The corneal grafts were completely clear after PK at 4 days on the right eye (C) and at 5 months on the left eye (D). In the left eye, the sutures had already been removed. After 3 years, both eyes (E, F) showed a clear corneal transplant in the center.

At the first presentation, the girl showed dense corneal opacity and limbal vascularization on both eyes. The opaque corneas of the girl precluded examination of deeper anterior segment structures (Figure 1A, B (Fig. 1)). Horizontal corneal diameter was 10 mm bilaterally, vertical corneal diameter was 9 mm on the right eye (R) and 8.5 mm on the left eye (L). Axial length was 17.28 mm (R) and 17.55 mm (L), anterior chamber depth was 2.05 mm (R) and 1.99 mm (L). The retina was attached on B-scan ultrasound in both eyes. The girl did not fixate or follow objects, and a conjugate horizontal pendular nystagmus as well as esotropia on the left eye were noted. Intraocular pressure (IOP) was normal (R 7.8 mm Hg, L 16.5 mm Hg by Schiötz tonometry).

A PK was performed on the left eye in April 2015 and on the right eye in September 2015 by an experienced corneal specialist (EB). Each trepanation of cornea of the recipient was 5.5 mm and each corneal transplant amounted to 6.0 mm with an endothelial cell density of ≥2500 cells/mm^2^, and was obtained from the Eye Bank Berlin, Charité – Universitätsmedizin Berlin. The first donor cornea was grafted with two continuous sutures using 10-0 and 11-0 monofilament nylon sutures (Resorba^®^, Nürnberg, Germany) and single sutures using 10-0 monofilament nylon (Resorba^®^, Nürnberg, Germany) on the left eye. The right eye was grafted with single sutures using 10-0 monofilament nylon sutures (Resorba^®^, Nürnberg, Germany) from the beginning.

Postoperatively, prednisolone acetate 1% (Pred Forte, Allergan, Irvine, CA, USA) and unpreserved ofloxacin 0.3% (Floxal^®^ EDO^®^, Bausch & Lomb, Berlin, Germany), both 5 times daily, were administered until the corneal sutures were removed on the left eye after 5 months, when the second keratoplasty surgery on the right eye was performed. Then, the treatment was changed to Loteprednoletabonat 0.5% (Bausch & Lomb, Berlin, Germany) 2 times a day. An epithelial defect of the left eye was treated with a bandage contact lens for four weeks. An anterior synechia at 4 o’clock and a loose suture were observed in the left eye one month after surgery. They were replaced, and the synechia was lysed with a spatula through a paracentesis. Throughout the postoperative course, the IOP was at a normal level, ranging from 11 to 18 mm Hg (iCare). The girl fixated large objects with her left eye after one month. One month after the second PK, the refractive error was +8.0 –8.0 x 135° (R) and +4.0 sph (L), so that glasses were prescribed. The anterior synechia was still present in the left eye, the lenses were clear, and the fundoscopic examination revealed a normal optic disc with a cup-to-disc ratio of 0.1, and a normal retina bilaterally. It was recommended that the girl wears the glasses and patches the left eye 1 hour a day. Five months after the second surgery on the right eye, all sutures were removed in both eyes. 17 months after surgery, visual acuity (VA) was 0.16 (Cardiff Acuity Test, CAT) on the left eye. A VA for the right eye could not be measured because the occlusion was not tolerated on the left eye. In April 2017, the girl could fixate and follow objects with the right eye; VA of the left eye was 0.4 with CAT. Complete anterior synechia were noted on the right eye, which were lysed surgically. Under general anesthesia, retinoscopy did not yield a sufficient reflex to measure the refractive error. Part-time occlusion of 1 hour a day of the left eye was continued.

Four years after the first operation, VA was 0.03 (LEA Vision Test in 1 meter) on the right eye, and 0.2 (LEA Vision Test in 6 meters) on the left eye. There was a right esotropia. Horizontal corneal diameter was 9 mm (R) and 10 mm (L), vertical corneal diameter was 10.5 mm bilaterally. Axial length was 21.13 mm (R) and 19.04 mm (L). IOP was 15 mm Hg in both eyes (iCare). The clinical presentation revealed well-adapted corneal transplants with a clear central graft in both eyes with otherwise normal anterior and posterior segment findings.

## Discussion

Sclerocornea is a primary anomaly of the eye in which the cornea blends with the sclera, having no clear boundary [14]. In our case, the mother of the child had a mutation in PITX3, a transcription factor containing homeodomain (HD) that causes cataract and anterior segment mesenchymal dysgenesis in several families [15], [16].

In general, corneal opacity such as Peters anomaly and sclerocornea have been considered as the main indication for PK in young children. However, there seem to be differences in regional clinical practice regarding the use of PK in this age group. The European corneal experts are more conservative and do not recommend PK in sclerocornea due to poor surgical outcomes [3], [4], [9], [17], in contrast to, for example, Asian colleagues [7], [10], [11], [12], [13].

To note, anatomic success after PK does not always correlate with visual outcome, because visual improvement depends on accurate optical correction and strict adherence to amblyopia therapy, if necessary [18]. The diagnosis and surgical treatment of a congenital corneal opacity may be challenging for several reasons. First, examination of the patient may be limited, necessitating an exam under general anesthesia. Glaucoma should be ruled out in any child with corneal opacities. Additional diagnostic modalities such as ultrasound of the anterior and posterior segment and anterior segment optical coherence tomography (OCT) may be required to identify the correct diagnosis. In young children, sclera and cornea are smaller and more elastic, and the anterior chamber is shallower, making watertight closure during a PK more difficult in children compared to adults [4], [10]. Increased vitreous pressure in children may result in protrusion of the iris-lens diaphragm when the globe is open during surgery. Postoperatively, pronounced inflammation may lead to synechiae and elevation of IOP [4]. However, the main complication is the graft failure, which is more common in children than in adults due to the difficulties of postoperative examinations. The reported graft survival rate varied from 32.6% to 78.6% in children for more than one year follow-up [12], [19]. Lin et al. revealed that in children aged 0 to 7 years, the transparent graft rate of congenital corneal opacities was about 55.6% during the 6- to 82-month follow-up period [13]. Michaeli et al. reported a graft survival rate of 78% in 38 children with congenital opacities with a mean follow-up of 40.4 months [19]. A study by Yang et al. demonstrated a graft clarity rate of 56% at 6 months and 44% at 3 years after PK in Peters anomaly [20]. A recent study by Zhang et al. showed a graft survival in 68.1%, with a mean follow-up of 33.7 months [10].

The main cause of corneal graft failure is irreversible rejection. Zhang et al. accounted a graft rejection in 33.8% of 160 eyes in infants and children, 52% occurred in regrafted patients [10]. Kim et al. emphasized that the graft survival largely depended on the type of congenital corneal anomaly [9]. In their study, 7 out of 8 grafts survived in patients with Peters anomaly, whereas 9 out of 12 failed in patients with sclerocornea [9]. These results were concordant with previous reports demonstrating a poor outcome of PK in sclerocornea [21], [19]. The difference is that Peters anomaly has a clear cornea in the periphery, whereas sclerocornea has scleral tissue extension and vascularization in the periphery of the cornea. This likely contributes to the poorer outcome of PK in sclerocornea and may be a key factor.

Our case also showed peripheral vascularization of the cornea. Postoperatively, the girl developed many risk factors for a potential rejection, including loosening of sutures, anterior synechiae, and new vascularization of the graft. However, there was no increased inflammation, fibrin reaction, or IOP elevation during the postoperative course. We had to change some loosened sutures, and for this reason, the fixation of the graft in children should be done by interrupted sutures as already recommended [3], [4].

Age may also be an important key factor regarding graft survival after PK in children. The infant’s immune system is still developing and is modulated in order to coexist with the mother’s immune system in a newborn for 3 months. While humoral immunity is transferred from the mother, T cell-mediated cellular immunity is being developed by the infant. Regulatory T cells (Tregs) have been described as T suppressor cells with a role of ocular privilege [22] and tolerance induction [23]. Two subsets of Tregs, natural Tregs (nTregs, which are developed in the thymus), and inducable Tregs (iTregs, which differentiate from naïve CD4 T cells in the periphery under the influence of particular environmental conditions) have been described. It is known that iTregs can induce tolerance in models of graft versus host disease and solid organ transplantation [24]. In an animal model, it was shown that subconjunctival application of naïve Tregs supports corneal graft survival in baby rats [25]. Further studies are needed to examine the immune system in children under 1 year of age after PK.

## Conclusion

In our case report, we presented a successful PK in a 3-month-old infant without rejections and a well-developed VA after 4 years. We think that in some individual cases a PK in children with sclerocornea is reasonable to prevent lifelong visual impairment.

## Notes

### Competing interests

DP discloses that she has financial relationships with Bayer and Allergan. EB discloses that he has financial relationships with Alcon, Johnson & Johnson, Zeiss, and Human Optics. MR and DJS declare that they have no competing interests.

### Scientist program participation

DP is participant in the Berlin Institute of Health (BIH) Charité clinician scientist program funded by Charité – Universitätsmedizin Berlin and BIH.
